# Is Strychnia Cumulative in the System?—Does It Affect the Encephalon?

**Published:** 1857-03

**Authors:** W. G. Thomas

**Affiliations:** Camden, New Jersey


*Is Strychnia Cumulative in the System?—Does it Affect the Encephalon?* By W. G. THOMAS, M.D. Camden, New Jersey.

As the above questions have of late been discussed *pro* and *con*, I shall, by your leave, contribute my mite in the way of facts, leaving theoretical discussion to others better qualified for it. The tincture of nux vomica has become quite a favorite remedy with me in cases of deficient innervation, as a general tonic, &c.; so much so, that I should be sorry were positive evidence found of its cumulative action. In my experience I have found no such evidence. I have given it in doses large enough to excite involuntary action of the muscles of the face and extremities for two, four, and six weeks continuously, and instead of the medicine showing an accumulative action, the patients almost invariably showed a diminution of susceptibility to the action of the remedy. I have always found the dose to be an experimental one; that is, I have nearly always had to feel my way upward from a minute quantity until its action was perceptible, the maximum dose varying very much in different individuals. I have not kept a regular account of my cases, but have some of them very vividly impressed upon my mind by some 'wonderful cures,' which I ascribed to the action of the tincture of nux vomica.

This day, Dec. 8th, I visited a patient whom I had under the use and the *influence* of the medicine since the 5th day of November last. To day I erased his name from my visiting list, he having been *cured* by the continuous use of the tincture of nux vomica for five consecutive weeks. The patient alluded to, a son of a Mr. F. is aged about eight years. I was called in to see him on the 22d day of October. He was down with typhoid fever, of that peculiar, low, intermittent form which has so strangely characterized this fever in Camdem and vicinity, this fall and winter, bidding defiance to antiperiodics, and yet differing very much from our ordinary typhoid fever. The case was a neglected one, the patient having been sick two weeks previous to my visit, and without regular medical attendance. The usual treatment was adhered to, modified by the peculiar state of the case, calomel, quinia, turpentine, diaphoretics, milk-punch, &c. with occasional use of astringents and opiates to check diarrhœa and lull pain in the bowels. There was evidently a diseased condition of Peyer's glands. He rallied in the course of a few days, and kept on improving gradually in strength, gained an excellent appetite, and became able to sit propped up in a chair. On the 5th day of November, his mother called my attention to the completely palsied condition of the lower extremities. He was not only unable to stand, but was unable to exercise any power over them; they obeyed only the power of gravitation, falling carelessly from side to side as the position of his body was changed. I ordered half an ounce of tinct. nux vomica in one ounce of white wine; ordered six drops three times a-day (equal two drops pure tinct. at a dose). But the medicine acted so violently, especially during sleep (causing the mouth to be drawn with a quick motion from side to side, the eyeballs to roll rapidly backward and forward, upward and downward, the hands to leap convulsively to the head), that I ordered the dose reduced to four drops (equal one and a third drops pure tinct.), three times a-day. This caused the same actions, but in a minor degree. As the system became less susceptible, the dose was gradually increased to twelve drops (equal four drops pure tinct.), three times a-day. The patient, after the lapse of ten days, was able to support his own weight for a few moments at a time upon his lower extremities. He kept on gradually improving, appetite voracious, and now, December 8th, upon my visiting him, he walked briskly backward and forward across the room without any staggering, or showing any sign of fatigue. Ordered the medicine gradually withdrawn.

In this case, you will perceive, instead of an accumulation there was a loss of power, twelve drops (equal four drops pure tinct.), at the end of five weeks, scarcely causing a twitch of the muscles, although one-third that amount, in the beginning, caused pretty violent contractions. Should any evil effects follow in this case, I shall feel bound to inform you.

Another case. Two years ago I was called in to attend to a

Mr. G. aged thirty years. Typhoid remittent. Recovered under the usual treatment. Improved rapidly in flesh; obtained full use of the upper extremities; but the lower extremities were powerless. I resorted to friction, &c. and various internal remedies for a week or ten days. I had never used strychnia, or any of the preparations of nux vomica, and was in fact afraid of it. I determined to try the tinct. nux vomica: gave it in ten drop doses (pure) three times a-day, gradually increased to thirty drops. When we reached the thirty drops (pure tinct. three times a-day), the contractions being quite perceptible, came back to twenty-six drops, but eventually returned to the thirty. In two weeks the patient was able to walk, run, and jump, and is now, and has been for the last two years, in the enjoyment of good health; though, I am sorry to add, he seems to think the pleasure of curing him should be my sole and sufficient reward. I might go on and show other cases similar to the foregoing, but these I presume will suffice.

*Does it Affect theEncephalon?*

I think it does. The boy, whose case I have first related, would invariably complain of headache, and become stupid and drowsy in fifteen or twenty minutes after taking the medicine, and would frequently, after taking the medicine, fall into a sound sleep, from which he would be awakened with some difficulty; the violent twitching of the muscles and rolling of the eyes inducing the mother to waken him. I have not seen so decided an effect upon the brain in any other case, yet I frequently have heard my patients complain of the medicine affecting their head. It could not be the small quantity of alcohol in the dose of twelve drops in the boy's case, for he was in the habit of drinking daily a goodly quantity of milk punch, in his fondness for which favorite medicine he would have commanded the admiration of a confirmed toper; and the drowsiness was shown only and regularly in fifteen or twenty minutes after taking the nux vomica. It would be well for some one to experiment upon some of the lower animals with minute doses of strychnia, with a view of ascertaining whether there be any cumulative action upon the system.—*Medical and Surgical Reporter.*

